# Implementation of a routine respiratory follow-up after prematurity less than 32 weeks gestation or below 1,500 g birthweight at preschool age—a two-year experience

**DOI:** 10.3389/fped.2025.1604037

**Published:** 2025-06-02

**Authors:** Michaela Höck, Anna Zschocke, Ulrike Pupp-Peglow, Carolin Marcher, Barbara Brunner, Maria Schütz, Ursula Kiechl-Kohlendorfer, Elke Griesmaier

**Affiliations:** ^1^ Department of Paediatrics II (Neonatology), Medical University of Innsbruck, Innsbruck, Austria; ^2^ Department of Paediatrics III (Cardiology, Pulmonology, Allergology and Cystic Fibrosis), Medical University of Innsbruck, Innsbruck, Austria

**Keywords:** implementation, preterm infants, prematurity, routine respiratory follow-up, preschool age

## Abstract

**Background:**

Growing evidence indicates that prematurity adversely affects lung function, even in early childhood, thus, a routine respiratory follow-up was implemented in our clinical setting. The aim of this study was to evaluate the acceptance of this examination and assess the feasibility of forced expiratory maneuvers and bronchodilator responsiveness test (BRT) in former preterm infants at preschool age and to present initial results.

**Methods:**

In November 2022, a respiratory follow-up was implemented for former preterm infants born at less than 32 weeks of gestation or with a birth weight below 1,500 g, who were born between 2016 and 2019 at Innsbruck Medical University Hospital. The evaluation included a standardized clinical examination, collection of medical history, spirometry, and a BRT.

**Results:**

A total of 107 former preterm infants (median gestational age 29.9 (28.1; 31.1) weeks and mean birthweight 1,250.5 (±355.6) grams performed spirometry. Successful spirometry was achieved by 93 (86.9%) children. Among these, 64 (59.8%) had normal pulmonary function and were symptom-free, however, ten (15.6%) showed a positive BRT. Twenty-nine children (27.1%) exhibited pathological test results and/or respiratory symptoms, with 13 (44.8%) of them testing positive for bronchial hyper-responsiveness. Fourteen children (13.1%) did not meet the quality control criteria for spirometry but were symptom-free.

**Conclusion:**

Our study demonstrated that a respiratory follow-up for preterm infants is highly accepted and feasible at preschool age. Up to 30% of infants were identified with impaired lung function and subsequently received appropriate management, highlighting the importance of standardized and routine respiratory follow-up for these children.

## Introduction

As survival rates improve due to advances in neonatal care, such as antenatal corticosteroids, exogenous surfactant therapy, and lung-protective mechanical ventilation, the long-term consequences of prematurity are becoming increasingly significant (1). Preterm infants, especially those with bronchopulmonary dysplasia (BPD), are particularly vulnerable to long-term respiratory diseases. BPD is strongly associated with lung function deficits and an increased risk of chronic obstructive pulmonary disease in adulthood (2–5). In response to this, a routine respiratory follow-up program for former very preterm children, including spirometry and bronchodilator responsiveness testing (BRT) at preschool age, has been implemented.

**Figure 1:**
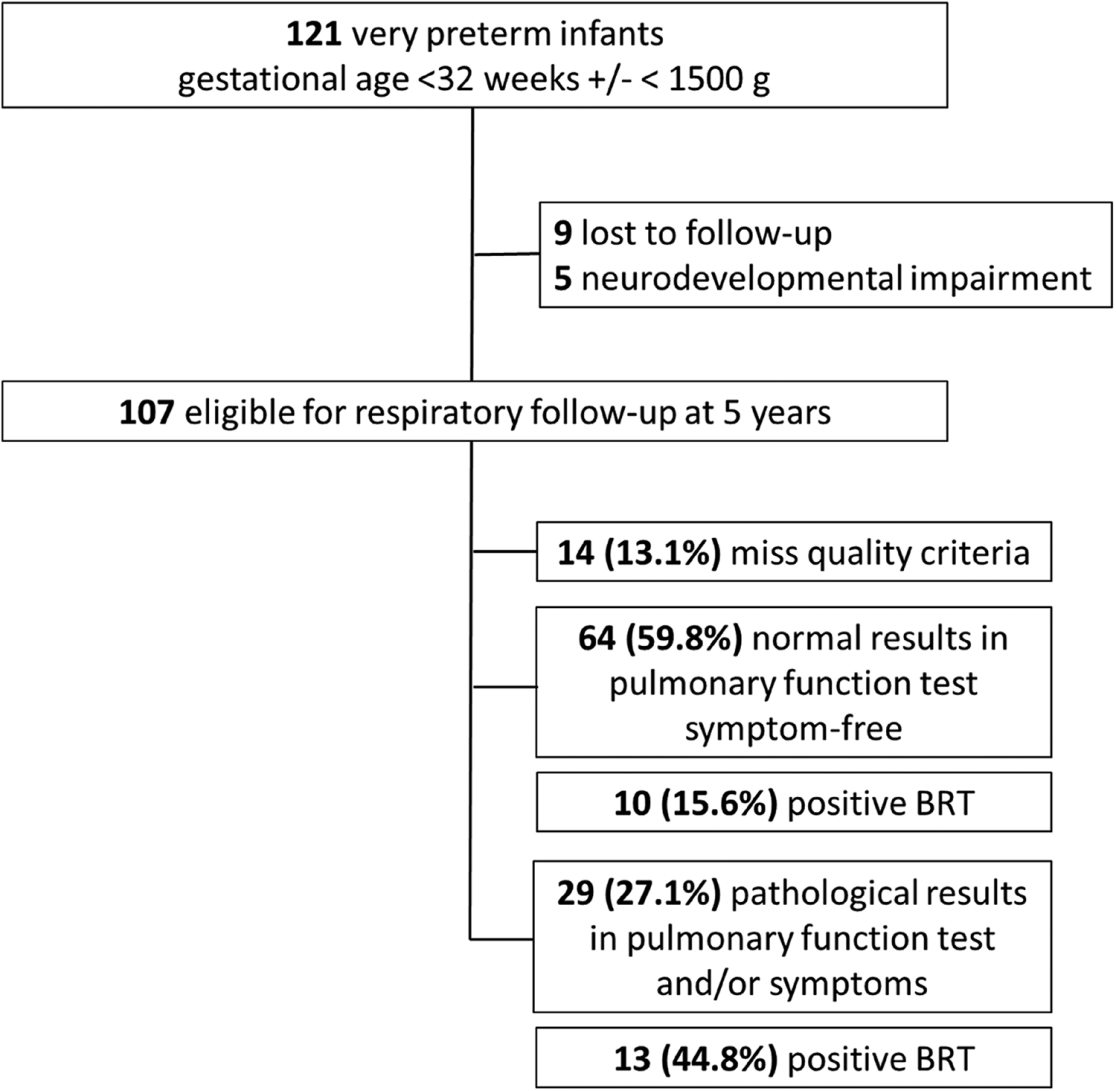
Flow-chart of enrolment and outcome of the study cohort. BRT, bronchodilator responsiveness test.

The aim of this study was to evaluate the program's acceptance and assess the feasibility of performing forced expiratory maneuvers and BRT in former very preterm infants at preschool age, and to present initial results.

## Methods

In November 2022, a routine respiratory follow-up program for former very preterm infants was integrated into the already established neurodevelopmental follow-up at preschool age for children born between 2016 and 2019.

### Participants

All infants born at <32 weeks gestational age or with a birth weight <1,500 g, who had been admitted to the Neonatal Intensive Care Unit (NICU) at the University Hospital of Innsbruck (the sole tertiary perinatal center in Tyrol) between January 2016 and December 2019, were eligible for inclusion*.* Data collection took place between November 2022 and December 2024. Children with severe neurological, cognitive, or motor impairments that would prevent them performing pulmonary function tests were excluded.

In this routine respiratory follow-up program, participants were assessed for pulmonary and atopic diseases, underwent a standardized clinical examination, provided a medical history survey, and performed spirometry, and a BRT. Perinatal and neonatal data were extracted from the medical records. Term control subjects were not recruited, as age-adjusted normal values for lung function tests are well-established (6–8).

### Operator details

A skilled operator is essential to obtaining high-quality lung function data (9). The operator, an experienced paediatric pulmonary nurse, ensured that the lips were sealed around the mouthpiece, the nose clip was in place, preventing any leaks during the maneuver. Encouragement, clear and simple instructions, demonstrations, and a stress-free environment were essential to help the children perform the maneuver effectively (10). The maneuver began with the child being instructed to “breathe in as deeply as possible”. Once the lungs were fully inflated, the child was instructed to “blow” the air out, followed by “more, more, more” until complete expiration. The child was encouraged to “keep going” even if they felt no further airflow. The operator visually inspected the performance of each maneuver to ensure quality before proceeding with the next. If the child showed signs of discomfort, the maneuver was immediately discontinued.

### Clinical and neurodevelopmental examination

Each child underwent a standardized clinical examination, which included cardiorespiratory assessments. Anthropometric data such as age (years), height (cm), weight (kg) (wearing indoor clothes with shoes), and body mass index (calculated as kg/m^2^) were recorded. Additionally, heart rate and oxygen saturation were measured using a pulse oximeter. The already existing neurodevelopmental follow-up program for former preterm infants focuses on the children's intelligence quotient (IQ) and was assessed using the Kaufman Assessment Battery for Children-II (K-ABC-II) or the Snijders-Oomen Nonverbal Intelligence Test (SON-R 2-8) as described previously (11).

### Medical history

A questionnaire based on the International Study of Asthma and Allergies in Childhood (ISAAC) study was used to gather information on respiratory morbidity after the neonatal period, preschool wheezing, recent asthma-like symptoms, and the use of asthma medication (inhaled corticosteroids, beta-2-agonists, or anti-leukotrienes) (12). Information on current and past smoking habits, pet exposure, and family history of atopy (defined as the presence of atopic diseases such as atopic eczema, allergic rhinitis, or asthma in a first-degree relative), were also recorded.

### Equipment

The spirometer used (Jaeger MasterScope spirometer, VIASYS Healthcare, Carl Reiner, Vienna) was capable of measuring instantaneous flow with an accuracy of ±5% and minimal dead space. The software featured incentive displays that encouraged rapid and prolonged expiration. The MasterLab system included interactive simulations, such as blowing up a balloon or blowing out candles, to assist in teaching and motivation.

### Spirometry

Lung function was measured using a spirometer, that met ISO 26782 standards with a maximum permissible error of ±2.5% when tested with a 3-L calibration syringe. Calibration was performed daily, and if the calibration factor deviated by more than ±2 standard deviations from the mean, this indicated the need for maintenance or repair of the spirometer, according to the manufacturer's instructions. Testing took place in a quiet, and child-friendly environment, separate from the waiting room and other patients. The examination was performed with the patient seated upright, the child's feet flat on the floor. Flow-volume measurements were compared to predicted values based on standard reference data from a European population, adjusted for age, sex, height, and ethnicity according to the Global Lung Function Initiative (GLI), as well as reference values from Zapletal et al. and the LUNOKID study (6–8). Impaired lung function was defined as a forced expiratory volume in one second (FEV₁), forced vital capacity (FVC), or FEV₁/FVC ratio below the lower limit of normal (LLN), which corresponds to z-scores < –1.64 (5th percentile), based on GLI reference values (13)*.*

### Bronchodilator responsiveness testing

Bronchial hyper-responsiveness (BHR) is a common feature of asthma, but can also be observed in individuals without respiratory complaints. Thus, a normal baseline spirometry does not rule out BHR, which is why BRT was conducted in all children to detect this condition (14). BRT measures the improvement in airflow after bronchodilator administration, indicated by a change in FEV1. After baseline spirometry, a β2-agonist, four doses of salbutamol (0.1 mg/dose) was administered via a spacer, and post-bronchodilator measurements were taken 10 min later. A positive response was defined as an increase in FEV1 of ≥12% from baseline (15). The average time required to complete both the spirometry and BRT was approximately 20–30 min.

### Quality of the examination

Quality control for preschool children's spirometry was based on ATS/ERS guidelines, with adjustments for developmental stage (16, 17). A successful test required an appropriate flow-volume curve with a sharp rise to peak flow, smooth descent, and acceptable FVC (6, 7, 18). Between eight and ten attempts, with adequate rest periods between each, were usually required for quality results.

### Data analysis and statistics

A retrospective analysis of prospectively collected data was performed using SPSS, version 29.0 for Windows (IBM Corp., Chicago, IL, USA). Descriptive statistics were used to characterize the individual variables and to determine the distribution of data, using the Shapiro–Wilk test. Values are expressed as numbers (frequencies, %), mean with standard deviation (±SD) and median with interquartile range (IQR). The Mann–Whitney *U* test, Student's *t*-test, and the *χ*^2^ test were used where appropriate. A *p*-value *p* < 0.05 was considered statistically significant.

### Compliance with ethical standards

The study was approved by the local Ethic Committee of Innsbruck Medical University (1013/2023) and adheres to the tenets of the Declaration of Helsinki.

## Results

### Characteristics of study cohort

A total of 121 former preterm infants were eligible for this study. Nine (7.4%) were lost to follow-up and five (4.1%) were unable to perform a spirometry due to severe neurodevelopmental impairment. Therefore, the study cohort consisted of 107 former preterm infants, Caucasian (European ancestry), with a male-to-female ratio of 64/43 (59.8%). The neonatal and characteristics at study visit are shown in Table 1.

**Table 1:** Characteristics of the study population.

	Entire study population (*n* = 107)	Patients’ subgroups		
		Normal results (*n* = 64)	Pathological results (*n* = 29)	*p* value
Neonatal characteristics				
Gestational age [weeks]; median (IQR)	29.9 (28.1; 31.1)	29.9 (28.3; 31.1)	30.0 (26.4; 31.2)	0.651
Birthweight [grams]; mean (SD)	1,250.5 (±355.6)	1,304.4 (±361.6)	1,204.0 (±345.2)	0.212
Male; *n* (%)	64 (59.8)	36 (56.3)	19 (65.5)	0.400
Bronchopulmonary dysplasia; *n* (%)	22 (20.6)	9 (14.1)	8 (27.6)	0.143
BPD mild; *n* (%)	15 (14)	8 (12.5)	5 (17.2)	0.373
BPD moderate; *n* (%)	4 (3.7)	1 (1.6)	2 (6.9)	
BPD severe; *n* (%)	3 (2.8)	0	1 (3.4)	
Patent ductus arteriosus; *n* (%)	18 (16.8)	11 (17.2)	4 (13.8)	0.638
Nectotizing enterocolitis; *n* (%)	4 (3.7)	2 (3.1)	1 (3.4)	0.965
Early-onest sepsis; *n* (%)	12 (11.2)	8 (12.5)	2 (6.9)	0.346
Late-onset sepsis,; *n* (%)	12 (11.2)	5 (7.8)	5 (17.2)	
Intraventicular hemorrhage; *n* (%)	15 (14)	8 (12.5)	5 (17.2)	0.577
Characteristics at study visit				
Height [cm]; mean (SD)	109.2 (±5.4)	109.0 (±5.6)	109.2 (±4.3)	0.838
Weight [kg]; mean (SD)	17.4 (±2.9)	17.5 (±3.2)	17.4 (±2.5)	0.880
BMI [kg/m^2^]; mean (SD)	14.5 (±1.5)	14.6 (±1.7)	14.5 (±1.2)	0.743
Age [years]; median (IQR)	5.1 (5.0; 5.3)	5.1 (5.0; 5.2)	5.1 (5.0; 5.2)	0.594
Oxygen saturation [%]; median (IQR)	99 (99; 100)	99 (98; 100)	99 (99; 100)	0.745
Heart rate; [bpm]; mean (SD)	97.9 (±12.5)	97.9 (±13.7)	97.1 (±10.8)	0.777
Intelligence quotient; mean (SD)	92.6 (±16.2)	92.8 (±13.8)	94.7 (±20.1)	0.583
Medical history				
Frequent infections; *n* (%)	32 (29.9)	12 (18.8)	19 (65.5)	**<0**.**001**
Respiratory morbidity; *n* (%)	30 (28)	15 (23.4)	12 (41.4)	0.143
Asthma medicaton; *n* (%)	42 (39.3)	22 (34.3)	17 (58.6)	**0**.**027**
Positive familiy history of atopy; *n* (%)	35 (32,7)	16 (25.0)	16 (55.2)	**0**.**002**
Smoking in pregnancy; *n* (%)	5 (4.7)	3 (4.7)	0	0.221
Smoking exposure; *n* (%)	22 (20.6)	13 (20.3)	4 (13.8)	0.493
Pets; *n* (%)	21 (19.6)	12 (18.8)	8 (27.6)	0.236

### Pulmonary function

At the time of the study visit, all children were free of clinically relevant symptoms and did not present with bronchitis or wheezing. Nine children (8.4%) had a mild upper airway infection (rhinitis). Cooperation was excellent, with all 107 children (100%) motivated to perform spirometry after their height, weight, and pulse oximetry were measured. Post-bronchodilator testing was performed in 88 children (82.2%), while the remaining children either refused the inhalation or the test did not meet quality criteria. Successful spirometry was achieved in 93 children (86.9%), with 64 children (59.8%) showing normal pulmonary function test results and being symptom-free. However, ten (15.6%) of these children had a positive BRT. Twenty-nine children (27.1%) exhibited a pathological pulmonary function test results and/or symptoms, of whom 13 (44.8%) had a positive BRT, and three (10.3%) were already under pulmonology care for recurrent and severe respiratory problems. Fourteen children (13.1%) did not meet quality control criteria but were symptom-free. The main reason for failure was poor effort due to lack of comprehension or coordination, rather than lack of motivation. The mean IQ of the children was 92.6 (±16.2), with the normal range defined as 100 ± 15. No significant difference in IQ was found between those who successfully completed the spirometry test and those who did not meet quality criteria (*p* = 0.196). The median number of attempts before the BRT was eight (IQR 6; 10), and seven (IQR 5; 9) after the bronchodilator inhalation.

Regarding respiratory history, 30 children (28%) reported previous respiratory issues, and 32 children (29.9%) reported frequent infections. Asthma-like symptoms and/or the use of asthma medication were reported in 42 (39%), while atopic dermatitis was observed in just 2 patients. Twenty-two children (21%) were exposed to smoking, 21 (20%) had pets at home, and 35 (33%) had a positive family history of atopy. The medical history is summarized in Table 1, and Figure 1 presents a flowchart illustrating the enrolment process and outcomes of the study cohort.

### Proposed process for clinical decision-making following initial pulmonary function testing

**Figure 2:**
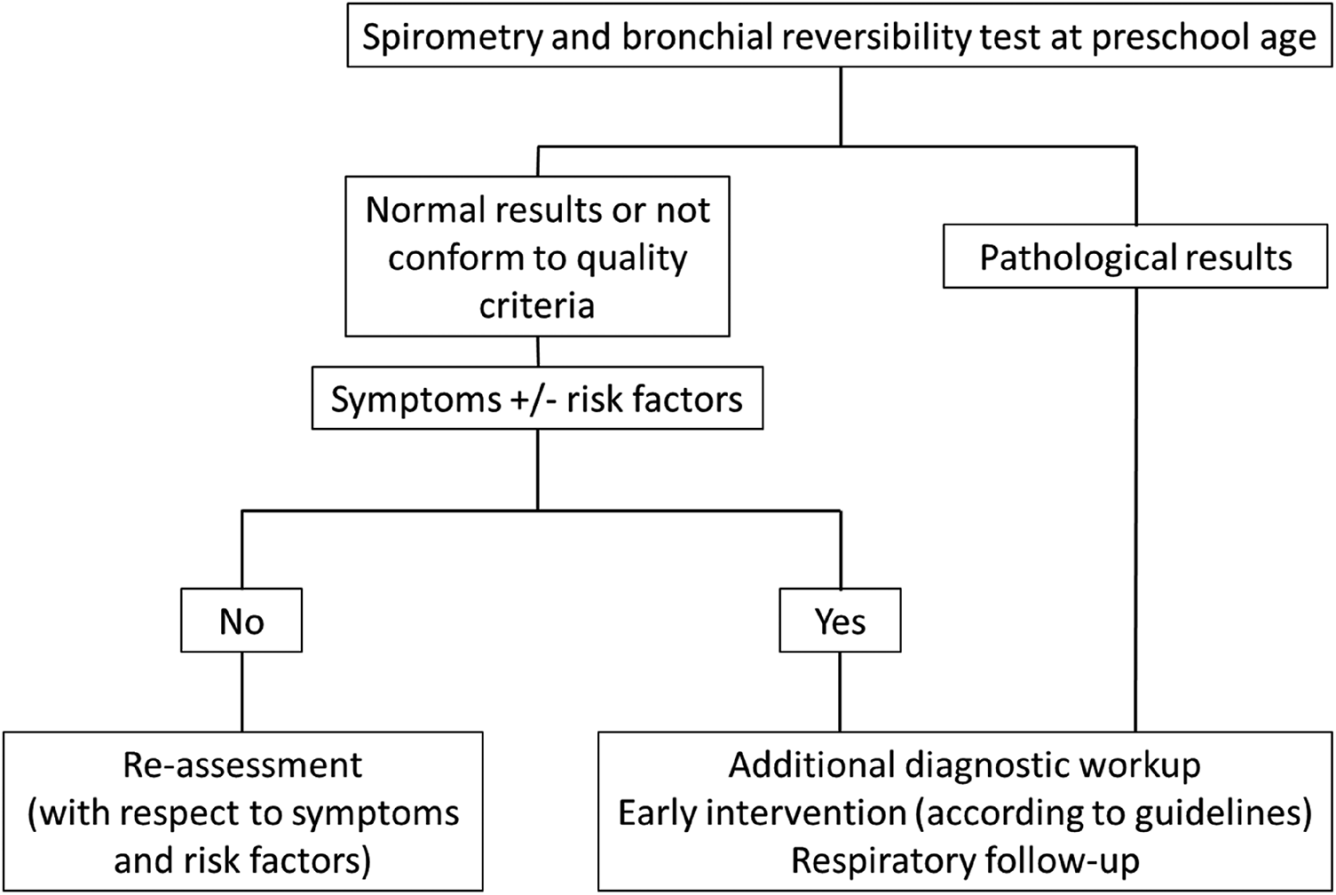
Proposed process for clinical decision-making following initial pulmonary function testing in former preterm infants.

If a child's first spirometry measurement was normal, no further pulmonary evaluation was planned, unless new symptoms or risk factors emerge. Detection of pathological spirometry results or a positive BRT should prompt early interventions. These interventions may include raising awareness about the importance of protecting the child's lung health, such as completely avoiding tobacco smoke exposure and improving indoor and outdoor air quality. Promoting beneficial health-care practices, such as recommended childhood vaccinations, proper nutrition, and encouraging physical activity can positively influence lung health. If necessary, appropriate medications may be initiated in accordance with current recommendations (19). Based on our experiences during this study, we developed a process to guide clinical decisions-making following initial lung function testing in former preterm infants at preschool age, shown in Figure 2 (20).

## Discussion

Prematurity, particularly when complicated by BPD, is a significant risk factor for long-term pulmonary function deficits (4, 21). Despite this, routine follow-up, including clinical assessments and lung function measurements, has not yet been universally established for these children. However, such a program is expected to improve clinical management and alleviate respiratory symptoms and airway obstruction. Spirometry is the most commonly used pulmonary function test, providing objective information for screening, diagnosing lung disease, and monitoring lung health. It enables the measurement of the impact of prematurity on lung function and the assessment of airway responsiveness (14). Additionally, spirometry is a non-invasive, well standardized, easy-to-perform tool, that offers clinicians essential information in conjunction with symptoms and physical findings.

When introducing a new method into clinical practice, it is essential to assess not only its acceptance but also the feasibility of forced expiratory maneuvers and BRT in former very preterm infants and interpret the results. Recent reports confirm that preschool children can perform technically acceptable spirometry (6–8, 18, 22, 23). In our study, 13% of children did not meet the quality control criteria, as spirometry requires active participation, and failure to follow instructions can lead to suboptimal results. It is therefore the operator's responsibility to engage the child and observe their performance to achieve optimal results. Testing former very preterm infants at preschool age typically takes 20–30 min and requires both time and patience. The child's developmental stage and IQ are important factors influencing their ability to perform the test. In our study, the children's mean IQ was 92.6, within the normal range (>85).

The study also aimed to assess the feasibility of performing forced expiratory maneuvers and BRT in former very preterm infants. Preschool children often have difficulty performing the forced expiratory maneuvers required for spirometry. They may be able to blow “hard” or “long”, but not both simultaneously. Even when FEV1 is achievable, its clinical value in this age group is questionable, as it may not accurately reflect bronchial obstruction. Previous studies have suggested using FEV0.5 or FEV0.75 as alternative outcome measures (24), as they are more clinically relevant at this age than FEV1 (24). Although we routinely used FEV1, exploring other spirometric parameters remains an important avenue for future research.

Defining acceptability criteria for spirometry results in preschoolers remains a crucial issue. High-quality pulmonary function data require three key elements: accurate and precise instrumentation, a patient capable of performing acceptable measurements, and an experienced nurse who can elicit maximum performance (9). As children become more familiar with the process and less intimidated, their performance often improve in subsequent sessions. This was observed in our study, where 60% of children had normal spirometry results and were symptom-free; however, 16% of them had a positive BRT. The overlap of measurements between healthy children and those with a history of wheezing makes the diagnostic accuracy of baseline pulmonary function tests generally poor. Therefore, BRT is recommended, as it provides a more reliable diagnostic profile than baseline lung function data alone. However, it is essential to differentiate between a positive BRT caused by true bronchodilation and one influenced by a learning effect from the second spirometry assessment. If the initial spirometry did not met acceptability criteria, a BRT was not performed. If the criteria were met, but the nurse observed that the child appeared more comfortable and confident during the second spirometry attempt, and suspected that the observed 12% improvement was due to a learning effect, this was documented, and no clinical decisions were based on the spirometry and BRT results.

Bronchial hyper-responsiveness is a common finding in children born preterm, but little is known about bronchial responsiveness in preschool children, and testing for bronchial hyper-responsiveness in this age group can be challenging (25, 26). In the general population it is known, that bronchial hyper-responsiveness increases with decreasing FEV1, atopy and smoking exposure, whereas a decrease is observed with increasing age (27, 28). In our population a careful observation of the child during the examination and a detailed medical history are crucial to avoid misinterpreting a “false” positive BRT as obstructive lung disease, or vice versa, which could lead to over or under-treatment.

It is also important to note that healthcare services in Tyrol, including follow-up care for former preterm infants, are free of charge and easily accessible. The low dropout rate in our study may be attributed to parents recognizing the benefits of monitoring their child's pulmonary health.

### Strength and limitation

Our study benefits from a well trained, multidisciplinary team of neonatologists, pulmonologists, and nurses, which is essential to ensure high-quality test results and to provide accurate and effective counselling. However, a single spirometry and BRT at preschool age do not allow lung function to be monitored and visualized over time. However, this tool has been easily integrated into the routine follow-up program for former preterm infants and represents an important step in enhancing awareness of their lung health status.

## Conclusion

Based on the findings of this study, which, to the best of our knowledge, is the first to report on the quality control of lung function assessments in former very preterm infants at preschool age, we demonstrated that respiratory follow-up is both highly accepted and feasible. Furthermore, we identified impaired lung function in up to 30% of former preterm infants that may have otherwise gone undiagnosed at this early stage. These results highlight the importance of standardized, routine respiratory follow-up for preterm infants and should serve as a valuable guide for clinicians.

### Directions for future research

Identify and analyze the causal factors associated with impaired lung function following preterm birth.

Develop and validate non-invasive methods for predicting impaired lung function in individuals born preterm.

Investigate the long-term outcomes of impaired lung function at preschool age after prematurity.

Establish evidence-based therapeutic strategies for the management of lung function impairment in former preterm infants.

## Data Availability

The original contributions presented in the study are included in the article/Supplementary Material, further inquiries can be directed to the corresponding author.
